# Extract from an Essay on Delirium Tremens

**Published:** 1846-07

**Authors:** 


*Extract from an Essay on Delirium Tremens.* By the late Lieut. Edwin Ramsey Long, M. D., *U. S. Army.*

The Illinois and Indiana Medical and Surgical Journal for June, 1846, contains the dissertation presented by our deceased friend, Lieut. Long, as a candidate for graduation at the Rush Medical College, Chicago, Illinois. We transfer to our columns the general conclusions at which he arrives from a comparison of the views entertained by different writers, and from his own observations and reflections on the subject. The disease was one which, from his connection with the army, he had large opportunities for witnessing, and of noting the effects of diversity of treatment. As our friend, Prof. Herrick, remarks in introducing the essay to his readers, it is "although unpretending in its character, intrinsically worthy of an attentive perusal, and will be especially acceptable to his numerous friends, who may wish to possess a memorial of one, estimable both as a companion, and an enthusiastic votary of science." The feature which to us is most interesting, and to which we would call the attention of our readers, is that philosophical tenor which recognises partial truth in facts apparently in opposition to each other, and avoids, both in precept and practice, exclusiveness of doctrine. This is eminently the characteristic of medical logic. Individual facts, or classes of facts, must not be considered alone, but in connection with others, and while the weight of each is to be carefully balanced, they are all to be adjusted and harmonised. In the instance of delirium tremens, numerous conflicting doctrines have been advanced respecting its pathology; each doctrine has been based on facts—veritable facts, but not necessarily true as involving in themselves the pathology of this affection. The writer of this essay has illustrated this in a very satisfactory manner, and by the method of exclusion (a method of very great practical availability in medical investigations) he reaches, by a short and conclusive process, the result most conformable to the present condition of our knowledge. The liability to errors of exclusiveness is not less in therapeutics than in pathology; the former being, to a great extent, a consequence of the latter. But there is certainly no maxim better established, than that every practical rule, however true in the abstract, requires in its application, modifications adapting it to circumstances peculiar to each case. This it is which renders the practice of medicine emphatically a rational art; involving the necessity of ratiocination even in the application of principles which have been empirically established. In this point of view, it appears to us, that without any bias from personal partiality, the dissertation from which we give the following extract contains an earnest of the success which would have attended the professional labors of our friend had his life been spared for science and humanity.—*Editor Buffalo Journal.*

From the foregoing extracts, which set forth the views of some of the most eminent authors, relative to the pathology and treatment of delirium tremens, I make the following deductions, viz:

1st. In *every* case there is found a peculiar morbid state of the sensorium commune, either dynamic or adynamic, manifested by delirium, tremors, sleeplessness and mental illusions.

2d. In a vast portion of the cases, decided evidences of gastritis, or other more serious lesions of the stomach, such as ulceration, scirrhus, &c., are found.

3d. That in other cases, where there are no marks of inflammation or ulceration of the stomach, there is noted a marked derangement of the digestive apparatus, such as foul tongue, nausea, &c.

4th. Not unfrequently traces of active inflammation of the brain, or its meninges, have been developed by autopsical researches.

5th. Torpor and impacted state of the bowels are not unusual, they being filled with feculent matter, and irritating secretions.

Such, then, being the prominent features of the disease, what is its pathology or essential nature? To determine this we must proceed on the principle of exclusion. It is, very obviously, an organic lesion of the stomach, or other part of the alimentary canal, or of the brain, or a peculiar morbid excitement of the nervous system, independent of inflammation in any tissues of the body.

Commencing with the first of them, we have it well authenticated, that cases of this disease which have terminated fatally, have been observed where no evidences of inflammation of the stomach were to be found, the same may be said of the intestines, and the brain, with its meninges; hence we conclude that the disease does not necessarily involve a lesion of either of these organs. But is there a case on record, in which the peculiar morbid excitement of the nervous system, as manifested by signs just enumerated, was not prominently and *invariably* presented? If not, are we not warranted in the conclusion, that this is the pathognomonic trait of the disease, without which it has no existence? All the other most prominent and common associations may be wanting, and still these phenomena are found, the former are not, therefore, essential parts of the disease, but may, and frequently do accompany it. For, although it may be said that a case of delirium tremens in which they are not found is extremely rare, yet *one* well established case, is of itself sufficient to demonstrate that these lesions are not essential to the disease; we, therefore assume that the malady is essentially one of the nervous system.

We are now ready to consider the various methods of treatment that have been most commended. In view of the facts that we have just exhibited, we have a disease of the nervous system, but most invariably complicated with inflammation of the brain or stomach, with torpidity and loaded condition of the intestinal tube, for which, some have recommended the exclusive use of stimulants, others, opiates, others, again, emetics, and a fourth class, equally strenuously advocating the antiphlogistic plan, not applying one or the other according to the varied symptoms of the case, but *one* and alone, single-handed, decrying all others as useless or decidedly pernicious. Such practice is too absurd to need comment. If, instead of pursuing this course, we adopt the more rational one of discarding all empiricism, and selecting remedies according to the varied features of the case, we will find that it is no difficult matter to determine upon a plan of treatment that will not disappoint our expectations in the result. In the first place, if we meet with a simple uncomplicated case of delirium tremens, as the disease is located in the nervous system, we have only to resort to such remedies as experience has found to be most effectual in controlling nervous symptoms, such as opiates, camphor, and the like. But as we seldom meet with such cases, but find them most usually connected with organic lesions of other tissues, we must primarily direct our attention to the investigation of these. By referring to the foregoing extracts, we see cerebritis, gastritis, and impacted bowels are the most frequent morbid associations, and consequently our first object is the removal of them, using for this purpose those remedial agents that are best suited to accomplish these ends.

As gastritis is the *most* common complication, with a full abdomen, we should make it a primary consideration to relieve the latter without aggravating the former. For this reason, we should not use cathartics, but give the preference to enemata, inasmuch as cathartics will inevitably *increase* the irritability of the stomach, and if inflamed, may occasion an incurable lesion of that organ. But in the event of there being a full, hard, tense pulse, bleeding should never be omitted, and if, superadded to this, there are marks of a strong determination of blood to the head, cups, or leeches will be advisable. Venesection, if practised, should precede other remedies. Should, however, a foul tongue, nausea, and other symptoms manifest a general derangement of the digestive organs, and no indication of gastritis be found, an emetic will do essential service. Having, then, premised venesection and emesis, when warranted by the symptoms just given, we will find no remedy so effectual as a moderately stimulating injection, by bringing away the vitiated matters lodged in the intestinal tube, which, perhaps, more than any other contingent disorder, tends to keep up the nervous irritability and mental hallucinations. Having cleared the bowels, we should next address the appropriate remedies to the stomach, to subdue any inflammation that may be indicated, such as iced water and leeches, bearing in mind, however, that we use not this, nor any other remedy, without a special indication by present symptoms, unless, perhaps, we have reason to suspect the existence of such disease that is masked by the predominant power of cerebral inflammation, and in this malady, as gastritis, is not unfrequently covered by inflammation of the brain, it may not be amiss to use some counter irritants or revulsions to the stomach, as a precautionary remedy.

After removing, by suitable remedies, all contingent or accidental disorders that we may find connected with the primary malady, which, as we have before stated, seems to be undoubtedly located in the nerves, we may now venture to administer such remedial agents as we have found to be most effectual in controlling morbid derangements of these tissues, such as opium, camphor, asafœtida, &c., and if we have been successful in the removal of what may be termed the collateral or contingent disorders, we will find that the primary or nervous malady will readily yield to this treatment, and our patient will soon fall into a refreshing sleep, that may be regarded as a favorable crisis of the delirium.

A moment's consideration of the several modes of treatment that have found most favor with practitioners, will show us at once why they have sometimes been successful, and at others totally inefficient or decidedly prejudicial. The truth is, that each of them is good in its place, and no one of them always applicable. It would be just as absurd to use opiates or stimulants invariably in all cases of this disease, as it would be to apply the like remedies to all cases of remittent fever, and precisely for the same reasons. We must discard all notions of empiricism, and be governed by established maxims of therapeutics, as much in this as in other maladies.

We are surprised to see that even Dr. Stokes seems to think that there are only two remedies necessary in this malady, one to remove gastritis, the other, the class of stimulants applicable to an opposite condition of the system. Now, I contend, that there are cases where it would not be proper to use either of them ; for instance, when the bowels are torpid and full of vitiated accumulations, what effect would iced water and leeches, to the stomach, on the one hand, or enormous doses of opium, on the other, have in subduing the nervous excitability? This is a case calling for enemata. Hence we see the propriety of selecting, in every case, such remedies as are indicated by present symptons.

From the foregoing, we conclude, that not the stimulating, the bleeding, the purging, or the antiphlogistic treatment is to be recommended *singly* or exclusively, but *each* or *all* of them as circumstances may render necessary and proper.

